# LEF1-AS1 accelerates tumorigenesis in glioma by sponging miR-489-3p to enhance HIGD1A

**DOI:** 10.1038/s41419-020-02823-0

**Published:** 2020-08-11

**Authors:** Zhihua Cheng, Guangyu Wang, Weiyi Zhu, Cong Luo, Zhilin Guo

**Affiliations:** 1 Department of Neurosurgery, Shanghai Ninth People’s Hospital, Shanghai Jiao Tong University, School of Medicine, No. 639 Zhizaoju Road, 200011 Shanghai, China; 2 Department of Neurosurgery, Shanghai Jing’an District Central Hospital, No. 259 Xikang Road, 200040 Shanghai, China

**Keywords:** CNS cancer, Cancer in the nervous system

## Abstract

Long non-coding (lncRNA) lymphoid enhancer-binding factor 1 antisense RNA 1 (LEF1-AS1) has been validated to be implicated in manifold cancers, whereas its function in glioma has not been understood thoroughly. Hence, in this study, we tested that LEF1-AS1 expression was significantly upregulated in glioma tissues and cell lines. Besides, knockdown of LEF1-AS1 repressed cell proliferation while activated apoptosis in glioma cells in vitro, and also suppressed tumor growth in vivo. RNA pull-down and luciferase reporter assays affirmed that LEF1-AS1 could bind with miR-489-3p. In addition, miR-489-3p expression was downregulated in glioma cells. Moreover, miR-489-3p depletion partly offset LEF1-AS1 knockdown-mediated function on proliferation and apoptosis. Further, HIGD1A identified as the target gene of miR-489-3p was upregulated in glioma cells. HIGD1A silence could restrict the process of glioma. In rescue assays, upregulation of HIGD1A remedied the inhibitory impacts of LEF1-AS1 silence on glioma cell growth. In summary, our studies corroborated the regulatory mechanism of LEF1-AS1/miR-489-3p/HIGD1A axis in glioma, suggesting that targeting LEF1-AS1 might be a promising method for glioma therapy in the future.

## Introduction

Glioma is a common brain tumor in adults, accounting for about 60%^1^. The distinct symbols of high-grade glioma contain neurocognitive impairment, poor quality of life as well as loss of life independence ability^2^. Although surgery has achieved great achievements in combination with radiotherapy and medication, the recurrence rate of glioma patients is still increasing^3,4^. Hence, it is essential to have a clear understanding of glioma and discover a brand-new target for treatment of glioma.

A number of literatures supported that long non-coding RNAs (lncRNAs) play an important role in the occurrence and development of various cancers, including glioma. For example, serum lncRNA HOTAIR serves as a new biomarker for the diagnosis and prognosis of glioblastoma multiforme^5^. LncRNA NEAT1 knockdown represses migration and invasion of glioma cells through regulating SOX2 targeted by miR-132^6^. Silencing lncRNA MIR22HG suppresses glioblastoma progression via inhibition of Wnt/β-catenin signaling^7^. The oncogenic role of LEF1-AS1 has been studied in prostate cancer and myeloid malignancy previously^8,9^. More importantly, LEF1-AS1 was abnormally upregulated in glioma tissues and closely associated with unsatisfactory prognosis of glioma patients via public database. Meanwhile, present research also confirmed that LEF1-AS1 was remarkably upregulated in tissues from glioma patient. However, the function of LEF1-AS1 in glioma has not been elucidated yet.

Emerging studies have highlighted the importance of competing endogenous RNA (ceRNA) network in multiple cancers. In this mechanism, lncRNAs functioned as sponges of microRNAs (miRNAs) to liberate mRNAs so that they could be released to code into proteins^10^. LncRNA NEAT1 and linc00152 exerted tumor-promoter role in glioma via modulating ceRNA network^6,11^. Therefore, we intended to investigate that whether LEF1-AS1 also played a role in glioma through the ceRNA network.

MiRNAs are a group of non-coding RNAs with 18-25 nucleotides in length, but without the protein-coding capacity^12^. MiRNAs are ubiquitous in almost all biological processes, including morphogenesis of metastasis, differentiation, and proliferation^13,14^. For instance, miR-103a-3p suppresses glioma cell proliferation and apoptosis^11^. MiR-124 signaling pathway affects cell proliferation, invasion and metastasis in glioblastoma multiforme^15^. MiR-423-5p is beneficial for enhancing the malignant phenotypes in glioblastomas^16^. In the present study, LEF1-AS1 expression mainly distributed in cytoplasm, indicating the post-transcriptional regulation potential of LEF1-AS1 in glioma. Further, bioinformatics suggested the binding relation between LEF1-AS1 and miR-498-3p, which was confirmed by the mechanical experiments. Nevertheless, the role of miR-498-3p in glioma was uncovered.

Moreover, the decisive role of protein-coding genes in cancer induced us to find out the target gene of miR-498-3p in glioma cells. Due to the most obviously regulatory effect of miR-498-3p overexpression on HIGD1A expression, HIGD1A was chosen as our researching focus. Therefore, whether LEF1-AS1 could regulate HIGD1A via miR-498-3p to affect glioma development was worthy exploring.

Overall, the main task of our study was to investigate the role of LEF1-AS1 in glioma. Besides, the regulatory mechanism of ceRNA network consisting of LEF1-AS1, miR-498-3p and HIGD1A was elucidated in glioma.

## Materials and methods

### Tissue samples

The matched glioma tissues and non-tumor tissues were collected from 46 glioma patients, with the approval from the Ethics Committee of Shanghai Ninth People’s Hospital and the informed consents from all patients. The participants didn’t receive chemotherapy or radiotherapy prior to surgery. Following frozen by liquid nitrogen immediately after surgical resection, tissue samples were kept at −80 °C for further analysis.

### Cell lines

Human glioma cell lines, including U251 (astrocytoma), T98MG (glioblastoma), SWO38 and U373MG (astrocytoma), and control cell line (HEB and NHA), provided by ATCC (Manassas, VA), were propagated in the DMEM (Invitrogen, Carlsbad, CA) under 37 °C with 5% CO_2_. The 10% FBS (Gibco, Waltham, MA) and 1% Pen/Strep solution were applied for supplementing DMEM.

### Quantitative real-time PCR (qRT-PCR)

Total RNAs extracted by TRIzol (Invitrogen) from U251 and T98MG cells were used for cDNA synthesis with the help of Reverse Transcription Kit (Toyobo, Osaka, Japan). SYBR Green Super Mix (Bio-Rad, Hercules, CA) was applied for qPCR experiment. Data were standardized to GAPDH or U6, and relative expression was calculated by 2^−ΔΔCT^ method.

### Transfection

The shRNAs of LEF1-AS1, HIGD1A and the relative negative control (NC) sh-NC, the miR-489-3p mimics/inhibitor and NC mimics/inhibitor, together with the pcDNA3.1/LEF1-AS1, pcDNA3.1/HIGD1A and NC pcDNA3.1, were procured from Genepharma (Shanghai, China) for transfection (2 µg/well). Lipofectamine 2000 (Invitrogen) was used for transfection of U251 and T98MG cells with indicated plasmids for 48 h.

### Colony formation assay

Transfected glioma cells were planted in triplicate in the 6-well plates (500 cells/well) for the 14 days of incubation at 37 °C. The colonies with more than 50 cells were fixed for 30 min in 4% formaldehyde (Sigma-Aldrich) and sequentially stained for 5 min by using 0.5% crystal violet (Sigma-Aldrich), followed by counting manually.

### EdU assay

EdU assay was performed in U251 and T98MG cells following the user guidebook of Cell-light™ EdU ApolloR567 in Vitro Imaging Kit (Ribobio, Guangzhou, China). Upon addition of fresh medium, EdU was added and cells were incubated for 2 h. After incubation, cells were washed by PBS to remove the medium containing free EdU. Next, cells were immobilized for 30 min in 4% paraformaldehyde (Sigma-Aldrich) before being stained by DAPI (Sigma-Aldrich), and then observed under fluorescent microscope.

### Flow cytometry for apoptosis

Annexin V apoptosis detection kit (Life technologies, Grand Island, NY) was employed for flow cytometry analysis. 2 × 10^5^ fresh glioma cells were reaped and put on ice for 1 h. Then, the resuspended cells in 100 µl binding buffer (Nanjing KeyGen Biotech Co., Ltd.) were processed with 5 µl PI (100 µg/ml) and 1 U/ml ribonuclease in a dark environment at room temperature for half an hour. Afterwards, cells were cultivated with 5 µl of Annexin V-FITC for additional 15 minutes, based on the manufacturer’s instructions. Subsequently, FACSCalibur (BD Biosciences, San Jose, CA) was utilized to analyze cells with an Attune flow cytometer (Thermo Fisher Scientific, Inc.). Software FlowJo 1.1.0 (Tree Star, Inc.) was used to determine the apoptosis rate.

### Terminal dexynucleotidyl transferase (TdT)-mediated dUTP nick end labeling (TUNEL) assay

Transfected glioma cells were subjected to treatment with 4% paraformaldehyde for 15 min and 0.25% Triton‐X 100 for 20 min. After that, cells were treated with TUNEL detection kit (Roche, Basel, Switzerland). Following DAPI dying, cells were analyzed under fluorescent microscope.

### Western blot

Total protein samples were subjected to electrophoresis on the 10% SDS-PAGE and then shifted onto the PVDF membranes. After sealing with the 5% skimmed dried milk, membranes were cultivated with primary antibodies against control GAPDH (ab8245; 1:1000), Bcl-2 (ab32124; 1:1000), Bax (ab32503; 1:1000), Total caspase-3 (ab13847; 1:500), Cleaved caspase-3 (ab49822; 1:500), and the appropriate HRP-tagged secondary antibodies (all, Abcam, Cambridge, MA). Protein signals were monitored by the detection system of enhanced chemiluminescence (ECL; Santa Cruz Biotechnology, Santa Cruz, CA, United States).

### Fluorescence in situ hybridization (FISH)

The RNA FISH probe designed for LEF1-AS1 was acquired from RiboBio and employed as per the standard method. After staining cells with Hoechst, images were captured by use of confocal microscope (OLYMPUS, Tokyo, Japan).

### RNA pull-down assay

The wild-type (WT) or mutated (Mut) sequences of miR-489-3p containing the putative LEF1-AS1 binding sites were biotinylated into Bio-miR-489-3p-Wt/Mut, which were further subjected to incubation with the cell lysates for 1 h. Finally, the beads were added to collect pull-downs and qRT-PCR analysis of indicated RNAs was followed.

### Dual-luciferase reporter analyses

The WT and Mut miR-489-3p binding sites within the LEF1-AS1 sequence or HIGD1A 3’-UTR were synthesized for inserting into the downstream of pmirGLO vectors (Promega, Madison, WI). The LEF1-AS1-WT/Mut and HIGD1A-WT/Mut were formed and co-transfected with indicated plasmids for 48 h, followed by the detection of luciferase activity via Dual Luciferase Assay System (Promega).

### RNA immunoprecipitation (RIP) assay

1 × 10^7^ glioma cells lysed in the RIP lysis buffer were prepared for the immunoprecipitation with beads conjugated to antibodies (Millipore, Billerica, MA) against control IgG or human Ago2. Precipitated RNAs were assayed by qRT-PCR.

### In situ hybridization (ISH) staining

ISH assay was performed to detect LEF1-AS1 expression in tissue samples. The experiment was conducted using the sensitive enhanced in situ hybridization kit of BOSTER Company (Wuhan, China) as required by supplier. The semi-quantitative ISH scoring standard was used to record the staining intensity and the number of positive zones. 4 grades, including 0 (negative), 1 (< 10% positive), 2 (10–50% positive) and 3 (> 50% positive), were used according to the staining proportion and intensity. The final scores were presented as low expression (0–1) and high expression (2–3).

### Xenograft model

Male BALB/c nude mice (6-weeks; weight: 20–25 g) were used for subcutaneous xenograft tumor experiment, with the approval of the Ethics Committee of Shanghai Ninth People’s Hospital. 1 × 10^8^ transfected cells were subcutaneously injected into nude mice, and tumor volumes were monitored every 4 days. Following 28-day of injection, mice were killed and tumors were carefully excised for weigh assessment.

### Hematoxylin and Eosin (H&E) staining

The collected tissues from xenograft model were fixed with 4% paraformaldehyde at 4 °C, embedded in paraffin and then cut into 4 µm sections. After de-paraffin, sections were rehydrated and stained with H&E (Sigma-Aldrich) at 4 °C for 10 min, finally observed under Olympus light microscope (magnification, ×200).

### Immunohistochemistry (IHC)

The tissue samples acquired in xenograft tumor assay were fixed and embedded in paraffin. After cutting, the 4 µm sections were subjected to IHC experiment using anti-Ki67 antibody (ab15580; 1:100; Abcam) in line with the standard protocol. After washing with PBS, the sections were incubated with HRP-conjugated secondary antibody (Abcam), and examined under light microscope (Olympus; magnification, ×200).

### Bioinformatics tools

The high expression of LEF1-AS1 in glioma tissues and prognostic significance of LEF1-AS1 for glioma patients were analyzed by GEPIA (http://gepia.cancer-pku.cn/) online public database. StarBase website (http://starbase.sysu.edu.cn/index.php) was utilized to predict the potential downstream miRNAs of LEF1-AS1 and the potential downstream target genes of miR-489-3p.

### Statistical analysis

Data analysis was developed by *t* test or one-way analysis of variance (ANOVA) using GraphPad Prism 6.0 (La Jolla, CA), with *p* < 0.05 as cutoff of statistical significance. Continuous variables of 3 or more independent assays were shown as the mean ± SD.

## Results

### LEF1-AS1 enhances the malignant growth of glioma cells

**Fig. 1:**
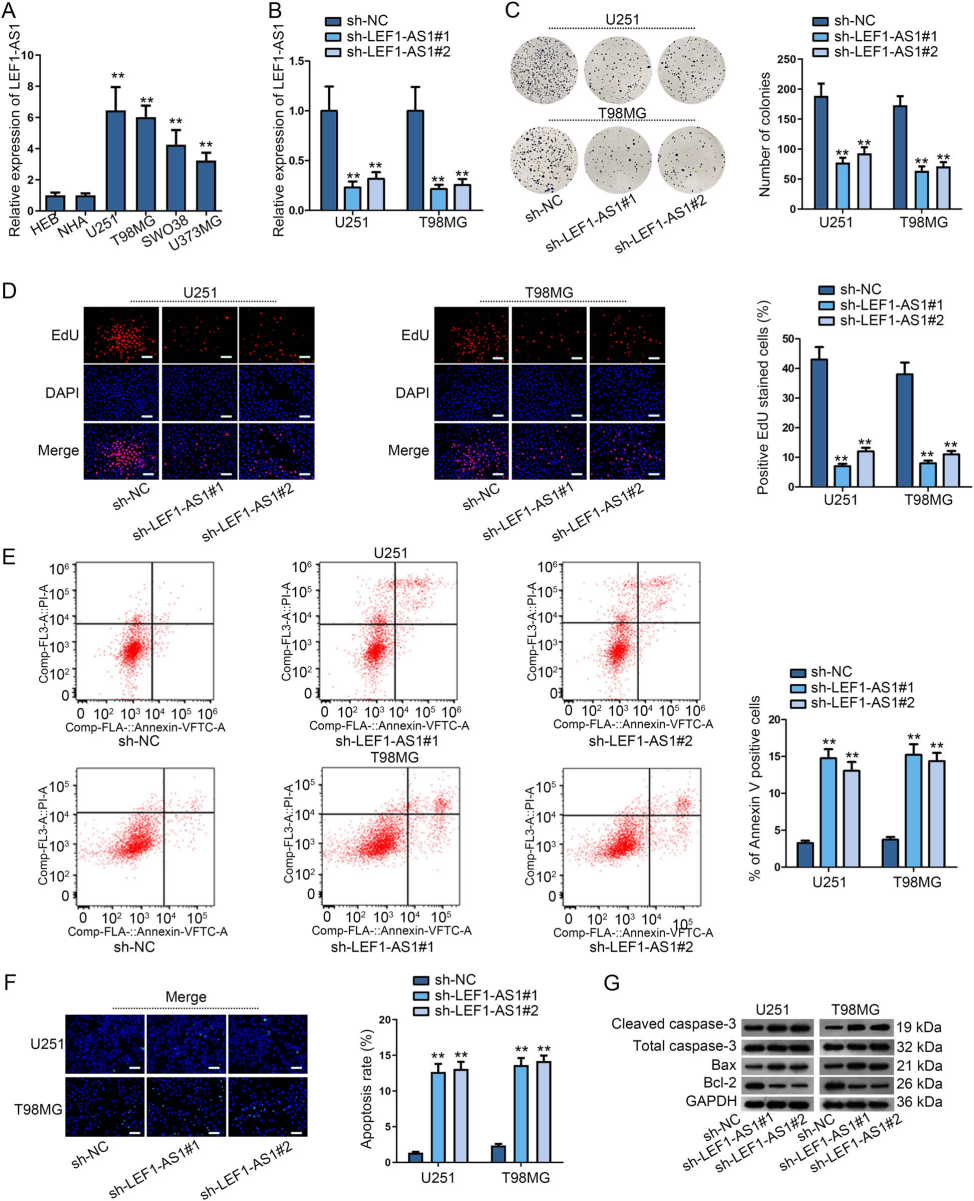
LEF1-AS1 enhanced the malignant growth of glioma cells. **a** LEF1-AS1 expression profile in glioma cell lines and normal HEB and NHA cells was measured by qRT-PCR. **b** LEF1-AS1 knockdown efficiency in U251 and T98MG was evaluated by qRT-PCR. **c**, **d** Proliferation of glioma cells was detected by colony formation and EdU (scale bar = 100 μm) assays when knocking down LEF1-AS1. **e**, **f** Flow cytometry analysis and TUNEL (scale bar = 100 μm) assay detected glioma cell apoptosis in response to LEF1-AS1 depletion. **g** Western blot was used to measure the expression of apoptosis-related proteins in glioma cells with or without LEF1-AS1 silence. **P < 0.01.

The previous study revealed that LEF1-AS1 was an oncogene in lung cancer. According to GEPIA public database, LEF1-AS1 was an aberrantly upregulated lncRNA in GBM tissues (*N* = 163) compared with normal tissues (*N* = 207) (Fig. S1a). Based on TCGA-GBM datasets, we found that high expression of LEF1-AS1 was closely related to short survival time of GBM patients (Fig. S1b), further indicating the potential contribution of LEF1-AS1 to the malignancy of glioma. Based on these results, we launched following investigations to probe into the precise role of LEF1-AS1 in glioma. Therefore, the expression pattern of LEF1-AS1 in glioma was measured via qRT-PCR firstly. Data delineated that LEF1-AS1 was obviously upregulated in glioma tissues and cell lines (U251, T98MG, SWO38 and U373MG) compared with non-tumor tissue groups and control cell lines (HEB and NHA), respectively (Fig. S1c and 1a). Besides, ISH assay further confirmed that LEF1-AS1 was highly expressed in glioma tissue samples compared with non-tumor tissue samples (Fig. S1d). Moreover, we unveiled that glioma samples from patients at high grades (WHO3-WHO4 grades) expressed higher LEF1-AS1 than those from patients at less malignant grades (WHO1-WHO2 grades) (Fig. S1e). To ascertain the biological function of LEF1-AS1 in glioma, we silenced LEF1-AS1 by sh-LEF1-AS1#1/2 transfection in U251 and T98MG cells, which contained the highest level of LEF1-AS1 among the indicated glioma cell lines (Fig. 1b). The colony formation assay revealed that LEF1-AS1 silence remarkably decreased glioma cell proliferation (Fig. 1c). Similarly, the proportion of positive EdU stained cells in sh-LEF1-AS1#1/2 transfected group was evidently less than that in sh-NC group (Fig. 1d). On the contrary, results of flow cytometry analysis exhibited an enhancement of glioma apoptosis in response to LEF1-AS1 depletion (Fig. 1e). TUNEL assay indicated that knockdown of LEF1-AS1 potently accelerated cell apoptosis in glioma as well (Fig. 1f). Consistently, suppression of LEF1-AS1 resulted in an increase of cleaved caspase-3 and Bax while a decrease of Bcl-2 (Figs. 1g and S1e). Thereafter, we also performed in vivo experiments through injecting sh-LEF1-AS1#1 transfected glioma cells into mice. As anticipated, tumor derived from LEF1-AS1-depleted cells looked smaller in size, along with a slower growth rate, than tumors originated from control cells (Fig. S2a). Also, we found the smaller volume and lighter weight of tumors with inhibited LEF1-AS1 than that of those in control group (Fig. S2b). Further, IHC staining assays elucidated that decreased Ki67 expression was observed in tumors with downregulated LEF1-AS1 (Fig. S2c). In brief, LEF1-AS1 downregulation conspicuously hindered glioma malignant growth both in vitro and in vivo.

### LEF1-AS1 sponges miR-498-3p in glioma cells

**Fig. 2:**
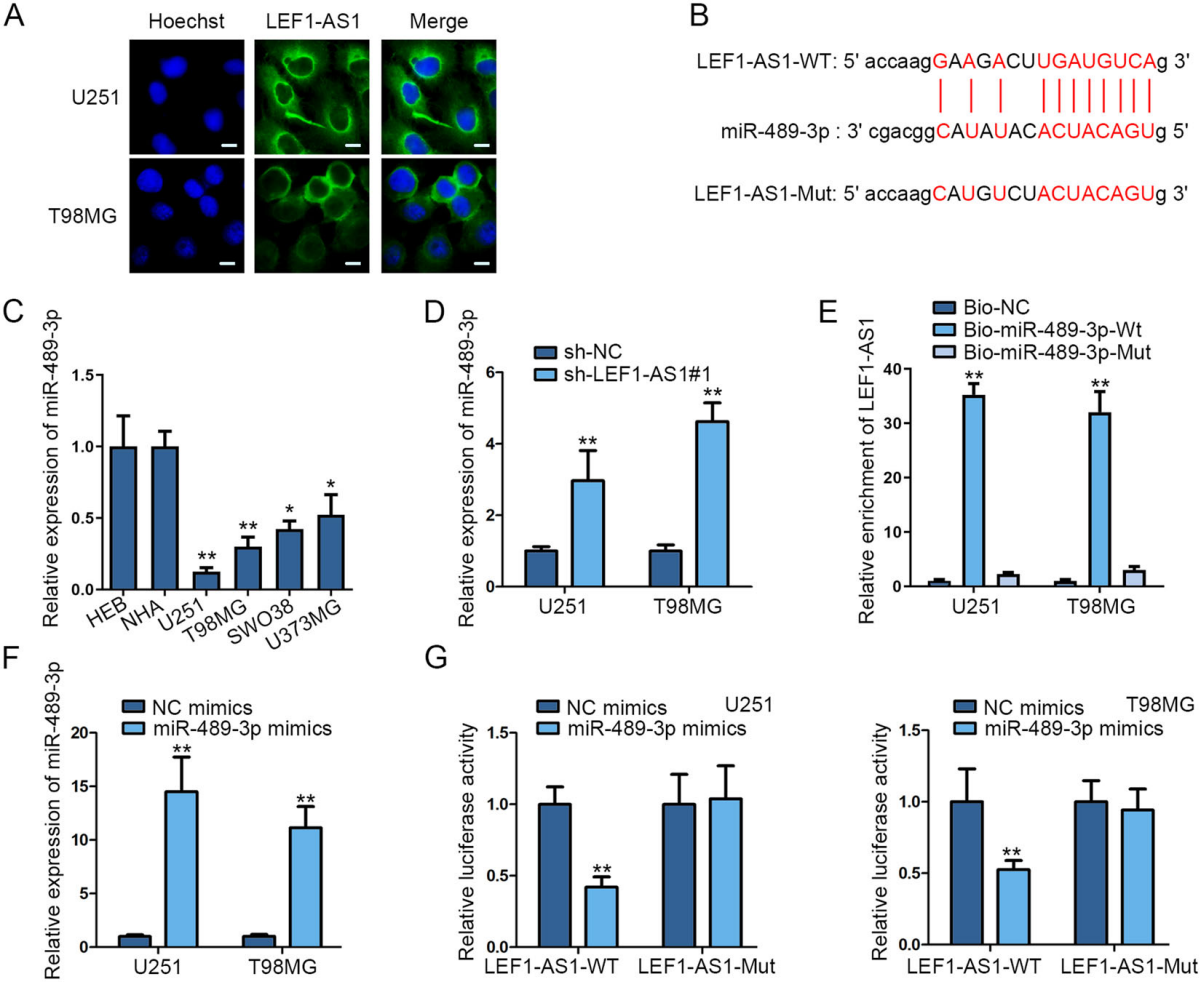
LEF1-AS1 sponged miR-498-3p in glioma cells. **a** Cytoplasmic location of LEF1-AS1 in glioma cells was detected by FISH assay (scale bar = 10 μm). **b** Bioinformatics (starBase) presentation of binding sites between LEF1-AS1 and miR-489-3p. **c** MiR-489-3p expression was detected by qRT-PCR in glioma cell lines and control cell lines. **d** MiR-489-3p expression was measured by qRT-PCR when downregulating LEF1-AS1. **e** RNA pull-down assay examined the interaction between LEF1-AS1 and miR-489-3p in glioma cells. **f** MiR-489-3p upregulation efficiency was examined by qRT-PCR in U251 and T98MG cells. **g** The luciferase activity of LEF1-AS1-WT/Mut was measured by luciferase reporter assay in NC mimics or miR-489-3p mimics transfected cells. *P < 0.05, **P < 0.01.

To investigate the regulatory mechanism of LEF1-AS1 in glioma, we firstly implemented FISH assay to detect LEF1-AS1 localization. Results confirmed that LEF1-AS1 was mainly distributed in cytoplasm of glioma cells (Fig. 2a). Hence, we guessed that LEF1-AS1 played its role via ceRNA pattern. Bioinformatics data obtained from starBase provided the binding site between LEF1-AS1 and miR-498-3p (Fig. 2b). Next, results of qRT-PCR unveiled that miR-498-3p expression was downregulated in glioma cell lines in comparison with normal controls (Fig. 2c). Thereafter, qRT-PCR unveiled the elevated expression of miR-489-3p in LEF1-AS1-silenced U251 and T98MG cells (Fig. 2d). Afterwards, RNA pull-down assay demonstrated that LEF1-AS1 was highly enriched in Bio-miR-489-3p-WT group not in Bio-miR-489-3p-Mut group (Fig. 2e). qRT-PCR assay illustrated that miR-489-3p expression was increased by transfecting miR-489-3p mimics into glioma cells (Fig. 2f). Subsequently, we observed that the luciferase activity of LEF1-AS1-WT was suppressed by miR-489-3p mimics while that of LEF1-AS1-Mut presented no notable changes (Fig. 2g). Altogether, LEF1-AS1 sponged miR-489-3p in glioma cells.

### MiR-498-3p depletion reversed the effects of LEF1-AS1 knockdown in glioma cells

**Fig. 3:**
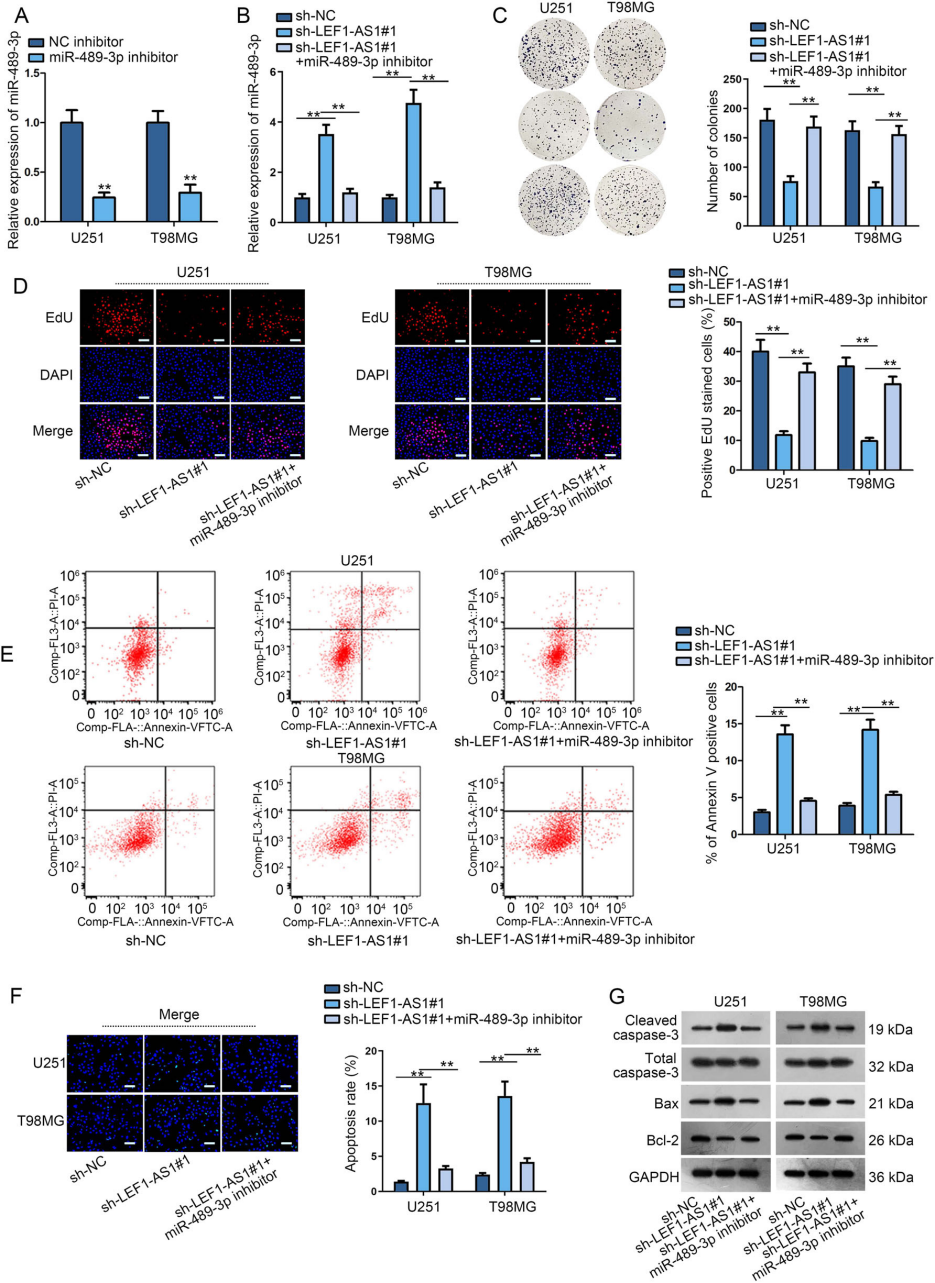
MiR-498-3p depletion reversed the effects of LEF1-AS1 knockdown on glioma cells. **a**, **b** MiR-489-3p knockdown efficiency and its expression in differently transfected groups were examined by qRT-PCR in U251 and T98MG cells. **c**, **d** Colony formation and EdU (scale bar = 100 μm) assays measured the proliferation ability of glioma cells transfected with sh-NC, sh-LEF1-AS1#1 or sh-LEF1-AS1#1+miR-498-3p inhibitor. **e**, **f** Flow cytometry analysis and TUNEL (scale bar = 100 μm) assay evaluated cell apoptosis ability under above conditions. **g** Western blot was performed to measure the expression of apoptosis-related proteins in indicated cells. **P < 0.01.

Through rescue experiments, we investigated whether LEF1-AS1 affected glioma cell proliferation and apoptosis via modulating miR-489-3p. MiR-489-3p expression was suppressed by miR-489-3p inhibitor in glioma cells (Fig. 3a). In addition, miR-489-3p expression was upregulated in sh-LEF1-AS1#1/#2 transfected cells, and then was normalized in cells co-transfected with sh-LEF1-AS1#1 and miR-489-3p inhibitor (Fig. 3b). In colony formation assay, we discovered that miR-489-3p depletion could offset the repressive influence of LEF1-AS1 downregulation on cell proliferation (Fig. 3c). Similarly, consequences from EdU experiments further verified the rescuing effects of miR-489-3p downregulation on LEF1-AS1 depletion-hindered cell proliferation (Fig. 3d). Additionally, the elevated cell apoptosis induced by LEF1-AS1 silence was recovered by miR-449b-5p inhibition, demonstrated by flow cytometry and TUNEL assays (Fig. 3e, f). Besides, the effects of LEF1-AS1 knockdown on cleaved caspase-3, Bax and Bcl-2 proteins were countervailed by miR-489-3p suppression (Figs. 3g and S3a). To sum up, LEF1-AS1 accelerated the progression of glioma via targeting miR-489-3p.

### HIGD1A silence curbed the malignant behaviors of glioma cells

**Fig. 4:**
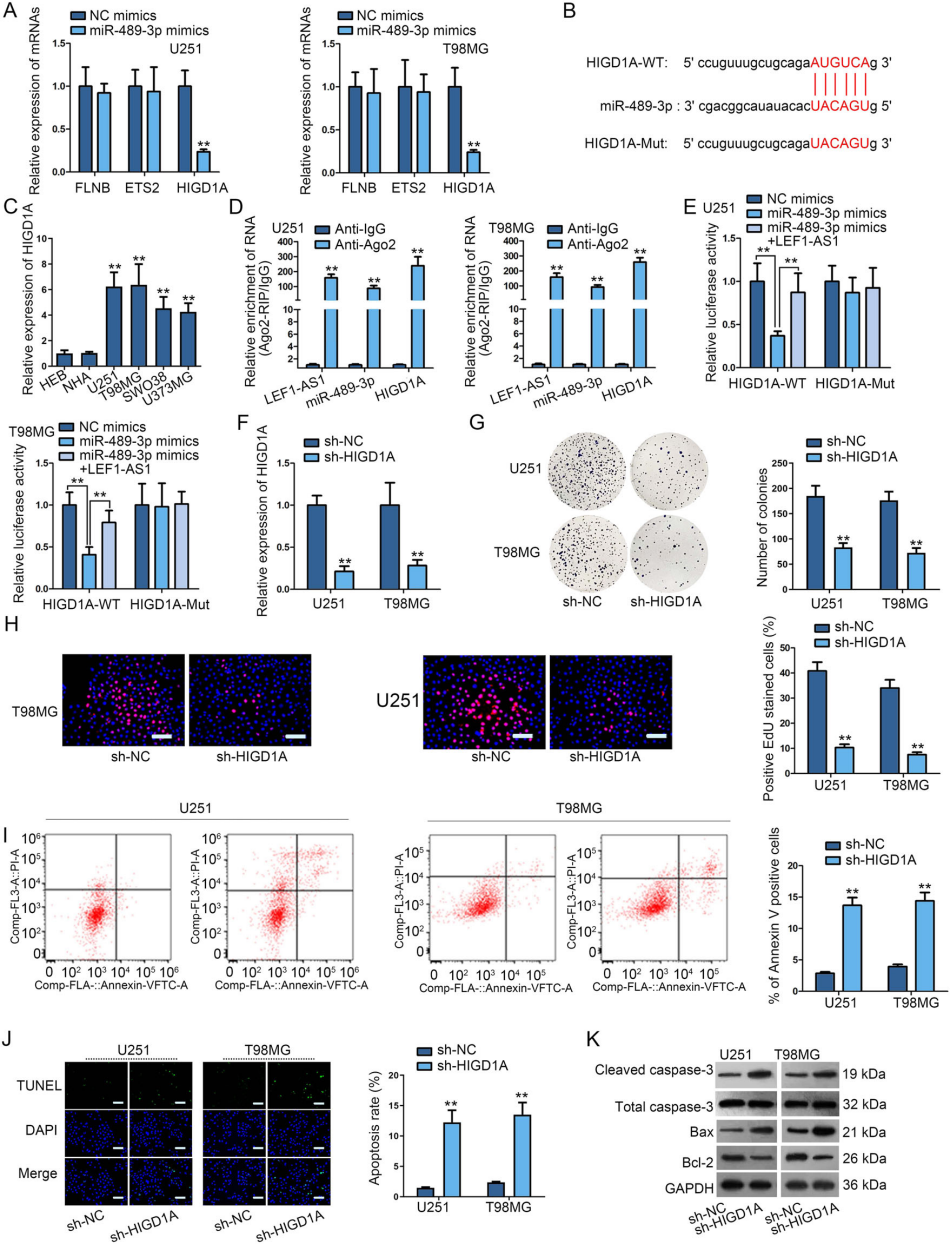
HIGD1A silence curbed the process of glioma cells. **a** The expression of FLNB, ETS2 and HIGD1A was measured by qRT-PCR when overexpressing miR-489-3p. **b** Bioinformatics (starBase) predicted the binding sites between miR-489-3p and HIGD1A. **c** HIGD1A expression was assessed by qRT-PCR in glioma cells and control cells. **d** RIP assay testified the enrichment of LEF1-AS1, miR-489-3p and HIGD1A in Anti-Ago2 or Anti-IgG group. **e** Luciferase reporter assay examined the luciferase activity of HIGD1A-WT/Mut in miR-489-3p mimics, miR-489-3p mimics+LEF1-AS1 or NC mimics group (**f**) HIGD1A knockdown efficiency was measured by qRT-PCR in U251 and T98MG cells. **g**–**j** The influence of sh-HIGD1A on glioma cell proliferation and apoptosis was measured by colony formation assay, EdU assay (scale bar = 100 μm), flow cytometry analysis and TUNEL assay (scale bar = 100 μm). (**k**) Western blot was conducted to evaluate the expression apoptosis-related proteins when knocking down HIGD1A. **P < 0.01.

Though searching starBase (http://starbase.sysu.edu.cn), FLNB, ETS2, and HIGD1A were screened out as the potential downstream target genes of miR-498-3p under the condition of strict stringency and high stringency. Then, the expressions of these genes were detected in glioma cells when overexpressing miR-498-3p. Prominently, HIGD1A expression was suppressed by miR-489-3p mimics while no obvious changes in FLNB and ETS2 expressions (Fig. 4a). Simultaneously, the complementary binding sites between miR-489-3p and HIGD1A were show in Fig. 4b. The expression of HIGD1A was tested in glioma cell lines (U251, T98MG, SWO38, and U373MG) and control cell lines (HEB and NHA). Results indicated that HIGD1A was highly expressed in glioma cell lines (Fig. 4c). Data of RIP assays disclosed that LEF1-AS1, miR-489-3p and HIGD1A were abundant in complexes precipitated by Ago2 antibody but not in compounds captured by IgG antibody (Fig. 4d). Later, it was revealed by luciferase reporter assay that miR-489-3p upregulation reduced the luciferase activity of HIGD1A-WT not that of HIGD1A-Mut, while such effect was then offset by upregulated LEF1-AS1 (Fig. 4e). Then, we examined the function of HIGD1A in glioma cells after validating the declined HIGD1A expression by sh-HIGD1A (Fig. 4f). It was revealed that HIGD1A depletion inhibited the proliferation and promoted the apoptosis of glioma cells (Fig. 4g–j). Meanwhile, HIGD1A knockdown inhibited the anti-apoptosis protein (Bcl-2) expression and stimulated pro-apoptosis protein (cleaved caspase-3 and Bax) levels (Figs. 4k and S3b). In a word, HIGD1A silence curbed the malignancy in glioma.

### LEF1-AS1 fostered the development of glioma via enhancing HIGD1A expression

**Fig. 5:**
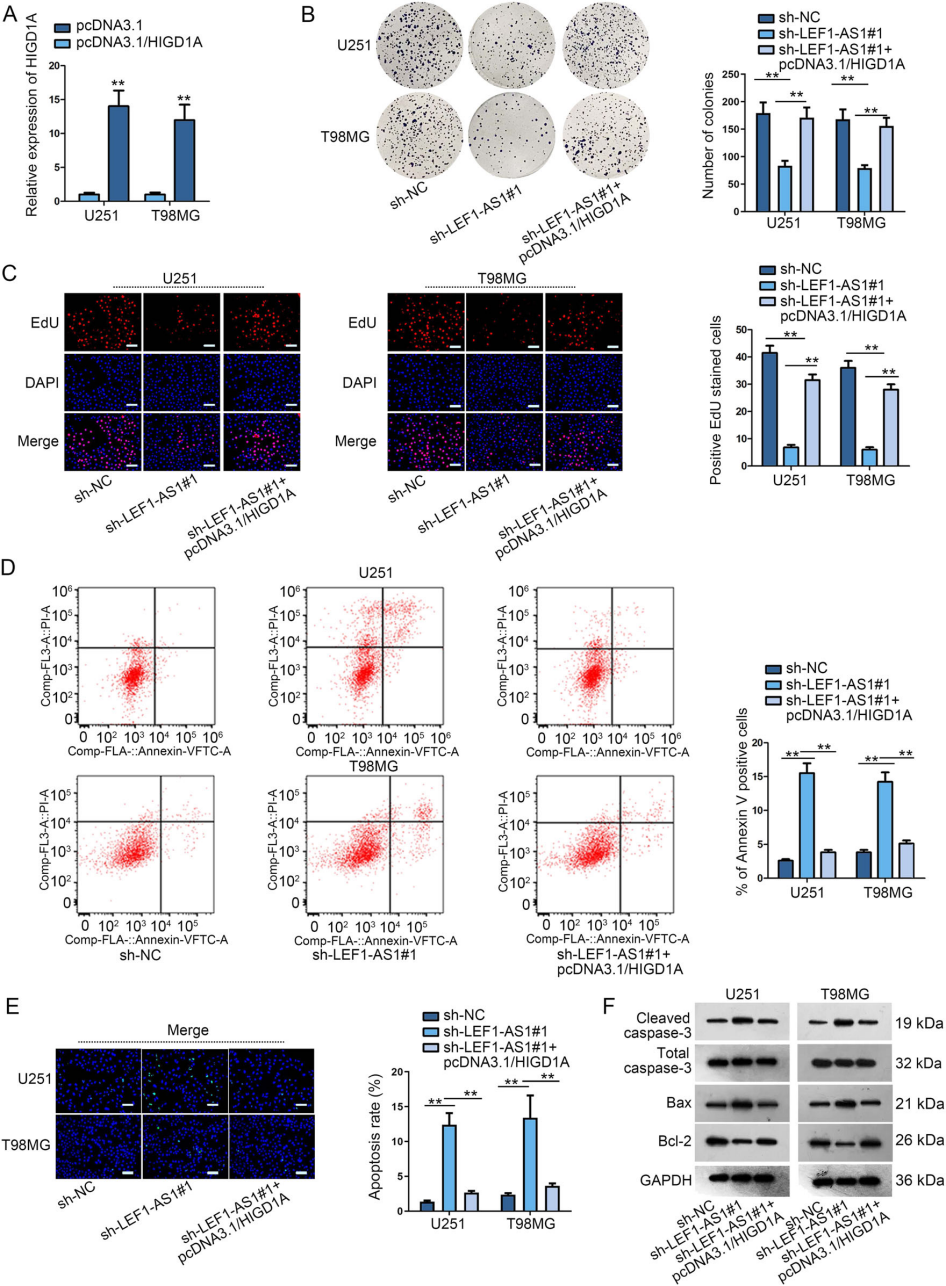
LEF1-AS1 fostered the malignant course in glioma via enhancing HIGD1A expression. **a** Overexpression efficiency of HIGD1A was validated by qRT-PCR. **b**–**f** The influence of sh-LEF1-AS1#1 or sh-LEF1-AS1#1 + pcDNA3.1/HIGD1A on glioma cell proliferation and apoptosis was assessed by colony formation assay, EdU assay (scale bar = 100 μm), flow cytometry analysis, TUNEL assay (scale bar = 100 μm) and western blot analysis. **P < 0.01.

**Fig. 6:**
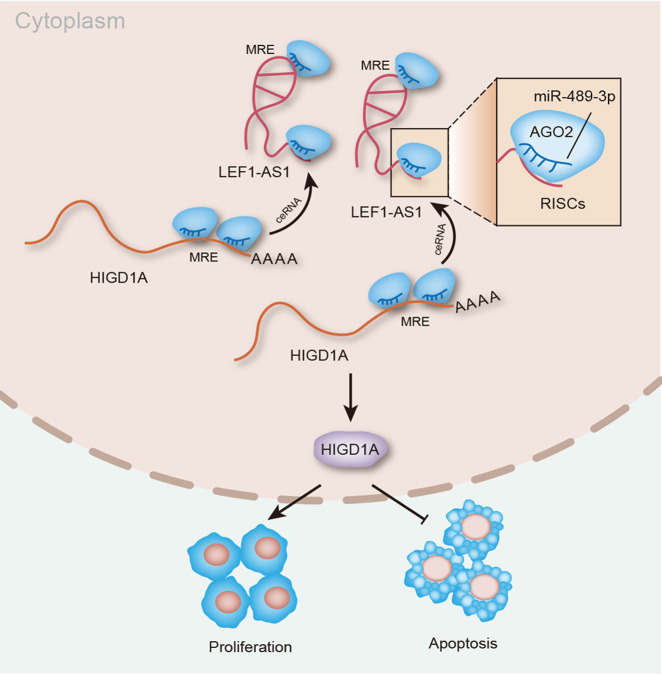
Graphical diagram. LEF1-AS1/miR-489-3p/HIGD1A axis in glioma.

Rescue assays were constructed to validate whether HIGD1A affected the modulatory effects of LEF1-AS1 on glioma cellular processes. To begin with, we confirmed the success overexpression of HIGD1A in cells transfected with pcDNA3.1/HIGD1A compared to those with pcDNA3.1 control (Fig. 5a). Colony formation and EdU assays testified that upregulating HIGD1A counteracted LEF1-AS1 silence-mediated suppressive function on cell proliferation (Fig. 5b, c). In terms of glioma cell apoptosis, the accelerating effects of LEF1-AS1 depletion on apoptosis was neutralized by augmented HIGD1A, suggested by flow cytometry analysis and TUNEL assay (Fig. 5d, e). Western blot pictured that the encouraging influence of LEF1-AS1 knockdown on cleaved caspase-3 and Bax expressions and suppressing influence of that on Bcl-2 expression could be counterbalanced by HIGD1A overexpression (Figs. 5f and S3c). In general, LEF1-AS1 facilitated glioma cell proliferation and inhibited cell apoptosis through function as a ceRNA to target miR-489-3p and thereby upregulated HIGD1A expression (Fig. 6).

## Discussion

In the late decades, the crucial influence of lncRNAs in glioma has been well-accepted such as its modulation on metastasis, chemoresistance and growth^17,18^. For example, H19 was described as an oncogene in glioma via inducing angiogenesis through repressing miR-29a^19^. LncRNA TUNAR was disclosed to suppress metastasis and proliferation in glioma via modulating miR-200a and Rac1^20^. MALAT1 regulated cell infiltration and treatment resistance in glioblastoma multiforme^21^. These findings provide insights into researching pathogenesis of glioma. Preliminary works reported that LEF1-AS1 worked as a potent oncogenic player in oral squamous cell carcinoma (OSCC)^22^. Noticeably, we examined that LEF1-AS1 was an upregulated lncRNA in glioma tissues and found that it was associated with short survival rate of glioma patients, which interested us to explore its role in glioma. LEF1-AS1 knockdown suppressed glioma cell proliferation in vitro and tumor growth in vivo, indicating LEF1-AS1 as a tumor facilitator in glioma.

Considering the cytoplasmic abundance of LEF1-AS1 in glioma cells and the vital role of cytoplasmic lncRNAs in ceRNA pattern, we unveiled the underlying interplay between LEF1-AS1 and miR-489-3p. Through experimental works, it was certified that miR-489-3p negatively regulated by LEF1-AS1, consistent with previous research findings^23,24^. In addition, miR-489-3p was lowly expressed in glioma cells. Furthermore, it was found that miR-489-3p depletion could restore the influences of LEF1-AS1 silence on glioma cell proliferation and apoptosis. Besides, miR-489-3p was identified as a tumor suppressor in renal cell carcinoma^25,26^ and bladder cancer^27^. Taken together, our study first shed new light on the relationship between LEF1-AS1 and miR-489-3p in glioma.

The regulatory effects of HIGD1A have been well-characterized in cellular survival and apoptosis in human carcinomas^28,29^. In this study, we uncovered that HIGD1A was the downstream target of miR-489-3p. In addition, HIGD1A was tested to be significantly upregulated in glioma cells. Depletion of HIGD1A led to decreased cell proliferation and activated apoptosis in glioma. Additionally, LEF1-AS1 knockdown-mediated function in the behaviors of glioma cells was offset by overexpressing HIGD1A.

In a word, our work elucidated that LEF1-AS1 interacts with miR-489-3p to relieve the suppressing effect of miR-489-3p on HIGD1A mRNA so as to increase the expression of HIGD1A, thereby promoting cell proliferation and inhibiting apoptosis in glioma, evidencing that LEF1-AS1 might serve as a potential therapeutic target for glioma patients.

## Supplementary information

Supplementary figure legends

Figure S1

Figure S2

Figure S3
