# Supplementary material for: LEF1-AS1 accelerates tumorigenesis in glioma by sponging miR-489-3p to enhance HIGD1A
Source: Cell Death Dis. 2020 Aug 11;11(8):690. doi: 10.1038/s41419-020-02823-0 (PMC7442828; doi:10.1038/s41419-020-02823-0)
Supplement: Supplementary file 1 — Supplementary figure legends [file 41419_2020_2823_MOESM1_ESM.docx]

**Supplementary figure legends**

**Figure S1** (A-B) Data of LEF1-AS1 expression in GBM tissues and its correlation with the survival rate of glioma patients were obtained from GEPIA database. (C-D) qRT-PCR analysis and ISH assay measured LEF1-AS1 level in glioma tissues and non-tumor tissues. (E) The expression of LEF1-AS1 in clinical tissues from glioma patients at WHO1-2 and WHO3-4 grades was evaluated by qRT-PCR analysis and ISH assay. (F) Data of two repeats of western blot analysis in Fig. 1G were exhibited, with the quantification diagram of blots in these three independent experiments provided accordingly. *P< 0.05, **P < 0.01.

**Figure S2** (A) The pictures of tumor obtained from mice injected with sh-LEF1-AS1#1/sh-NC transfected glioma cells were taken, along with the corresponding tumor growth curves. (B) The volume and weight of tumors obtained in above two groups. (C) IHC staining assays exhibited Ki67 expression in tumors from two different groups. **P < 0.01.

**Figure S3** (A-C) Related data of two additional experiments of western blot assays in Fig. 2G, 4K and 5F were exhibited. Besides, the quantifications of western blot were provided accordingly. **P < 0.01.
