# Supplementary figures and images for: LEF1-AS1 accelerates tumorigenesis in glioma by sponging miR-489-3p to enhance HIGD1A
Source: Cell Death Dis. 2020 Aug 11;11(8):690. doi: 10.1038/s41419-020-02823-0 (PMC7442828; doi:10.1038/s41419-020-02823-0)

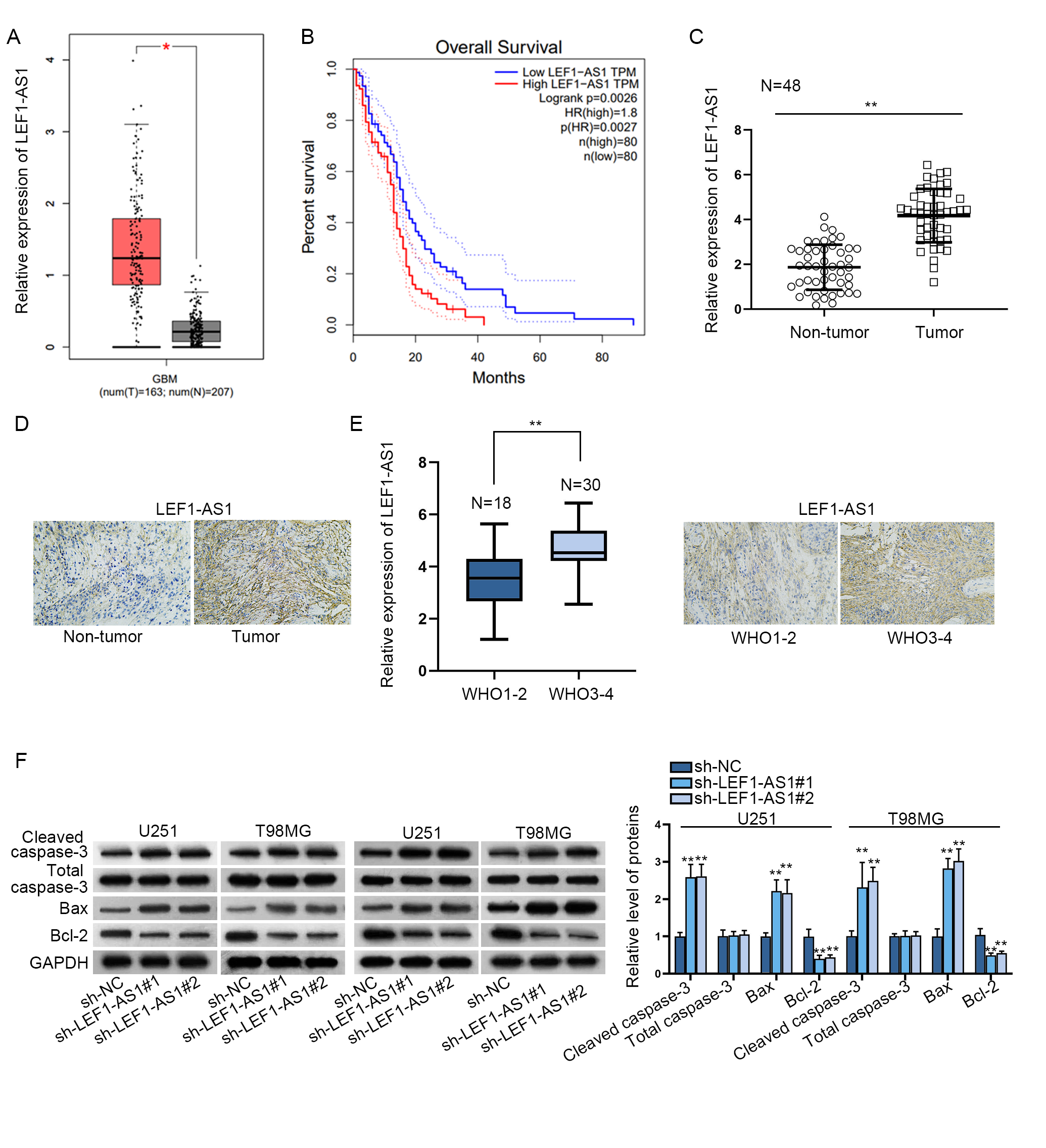

Supplement: Supplementary file 2 — Figure S1 [file 41419_2020_2823_MOESM2_ESM.tif]

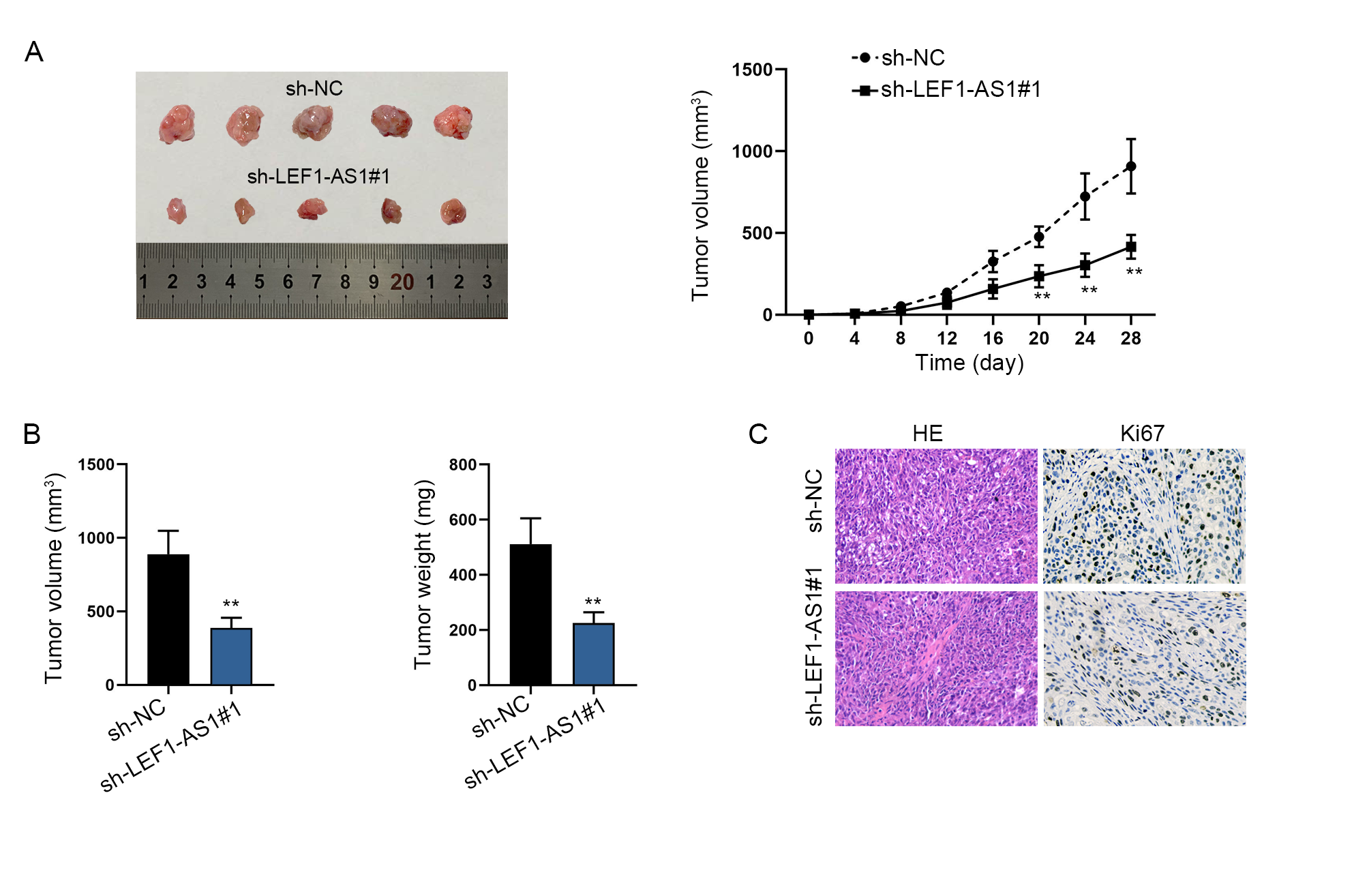

Supplement: Supplementary file 3 — Figure S2 [file 41419_2020_2823_MOESM3_ESM.tif]

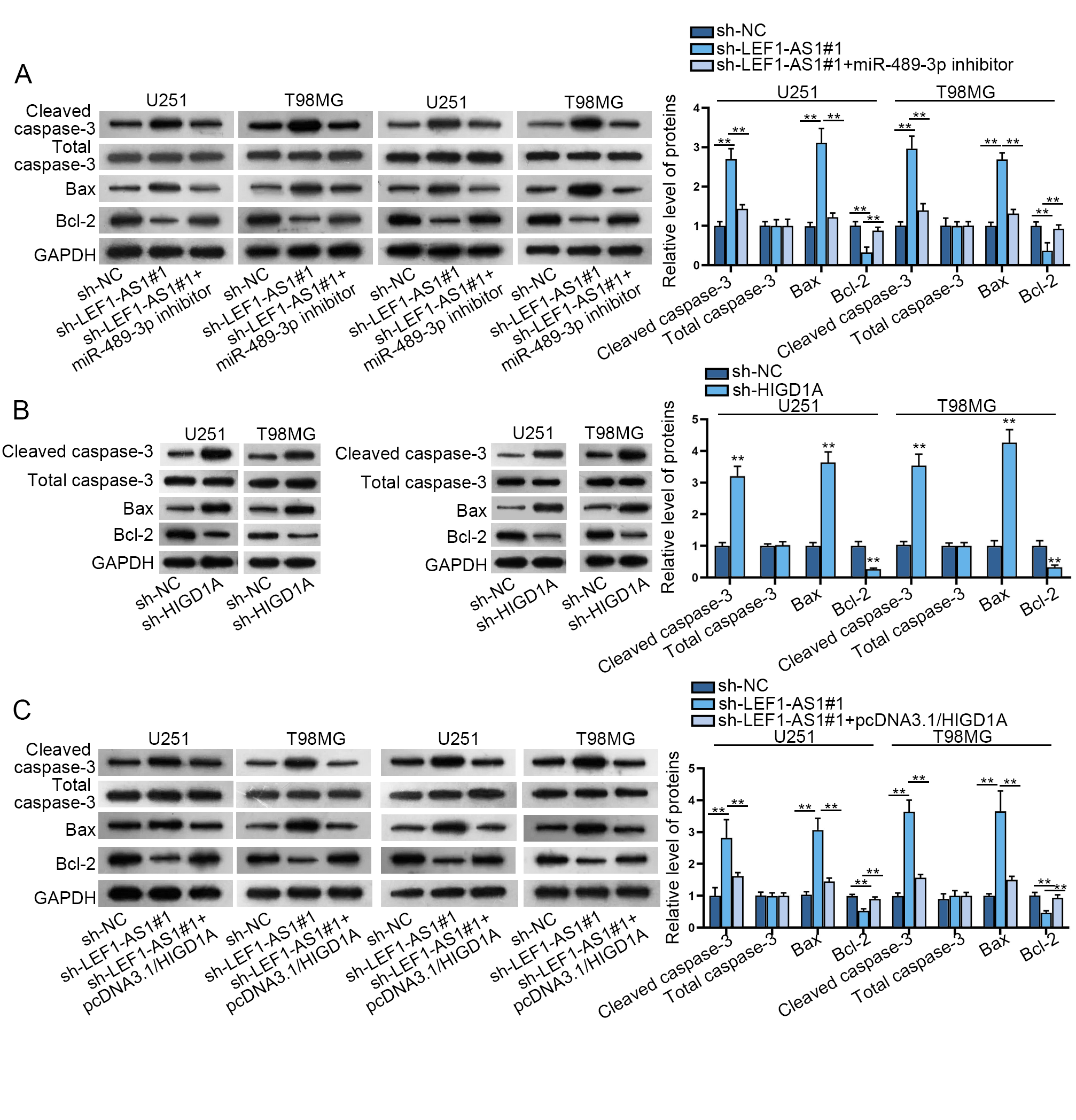

Supplement: Supplementary file 4 — Figure S3 [file 41419_2020_2823_MOESM4_ESM.tif]
